# Cardiac-Related Lesions in Newly Diagnosed Patients With Acute Leukemia: A Chinese Population-Based Real-World Study

**DOI:** 10.3389/fmed.2022.844350

**Published:** 2022-06-09

**Authors:** Wei Xiao, Linlu Ma, Yufeng Shang, Fuwei Yang, Yuxin Tan, Guopeng Chen, Jinxian Wu, Yuxing Liang, Tuerxunayi Rouzi, Qian Wang, Nan Zhang, Fuling Zhou

**Affiliations:** Department of Hematology, Zhongnan Hospital of Wuhan University, Wuhan University, Wuhan, China

**Keywords:** acute leukemia, cardiac damage, hyperleukocytic leukemia, myocardial enzyme, echocardiography

## Abstract

The relationship between newly diagnosed acute leukemia (AL) and heart-related lesions remains unclear. This study aimed to investigate baseline cardiac function and risk of cardiovascular diseases (CVDs) in patients with new-onset AL, and provide data on cardiac management strategies for patients with AL. We retrospectively collected data on baseline characteristics, echocardiography, and biochemical blood indicators (e.g., myocardial enzymes) from 408 patients, 200 with newly diagnosed AL, 103 with coronary artery disease (CAD), and 105 controls from January 1, 2015 to August 31, 2019. The creatine kinase isoenzyme myocardial band, lactate dehydrogenase, highly sensitive troponin-I, and B-type natriuretic peptide levels and left ventricular internal diameter (LVID) were significantly higher in patients with newly diagnosed AL than in the control group. The degree of cardiac damage was lower in newly diagnosed AL patients than in CAD patients. The best predictor of heart damage was LVID (AUC [area under the curve] = 0.709; 95% CI [confidence interval]: 0.637–0.781; *p* < 0.001), and independent prognostic risk factors were age and ejection fraction (HR [hazard ratio] = 1.636; 95% CI: 1.039–2.575; *p* = 0.033). The ratio of leukemia blasts among patients with AL was positively correlated with cardiac damage. Our data indicated that newly diagnosed AL patients had certain myocardial damage before treatment. Clinicians need to pay attention to these manifestations, which may be related to the prognosis.

## Introduction

Heart disease and cancer are the two leading causes of global mortality (1). Worldwide, 17.9 million deaths were due to cardiovascular diseases (CVDs) in 2019, accounting for 32% of global deaths (2). Nearly 10 million deaths were due to cancer in 2020 globally, and approximately 70% occurred in low- and middle-income countries (3). Leukemia is a life-threatening malignant clonal disease of the hematopoietic system and plays an important part in the global cancer burden (4). According to the 2017 Global Burden of Disease study data, the number of newly diagnosed leukemia cases increased from 354,500 in 1990 to 518,500 in 2017. Globally, the number of acute myelocytic leukemia (AML) deaths rose to 92.98% from 1990 to 2017, while acute lymphoblastic leukemia (ALL) deaths increased by 40.12% (5).

In recent years, researchers have paid increasing attention to the new field of cardio-oncology (6, 7). Studies have found that cancer survivors have an increased risk of CVDs, mostly due to the cardiotoxicity of anti-cancer agents (8–10) or shared common lifestyle risk factors (11, 12). In addition, cancer and CVDs share common systemic pathogenic pathways and mechanisms: inflammation, metabolic modification, clonal hematopoiesis, and altered angiogenesis, as well as extracellular matrix (ECM) and stroboscopic cells (13). Therefore, cancer often coexists with cardiac-related lesions. Studies have found that secondary cardiac tumors (often malignant) with a high incidence most often metastasize from the respiratory system, followed by the hematopoietic system (leukemia/multiple myeloma) (14). The clinical manifestations of leukemic infiltration of the heart include congestive heart failure, pericardial effusion, infiltrative cardiac disease, and constrictive pericarditis (15, 16). Due to high cytokine release or direct leukemic myocardial infiltration, patients with AL may present cardiac dysfunction before starting anthracycline chemotherapy (17). Moreover, hyperleukocytic leukemia, associated with increased white blood cells, greatly increases the incidence of acute myocardial infarction (AMI) and stroke (18).

This study aimed to investigate baseline cardiac function and risk of CVDs in patients with new-onset AL, explore CVD risk factors present before treatment and their association with prognostic survival, and provide data on cardiac management strategies for patients with AL.

## Materials and Methods

### Study Design and Patients

This single-center retrospective observational study included patients with newly diagnosed AL, CAD and controls seen at Zhongnan Hospital, Wuhan University from January 1, 2015 to August 31, 2019. All study participants were Chinese and ≥ 18 years old. The AL group included patients seen at the department of hematology who were newly diagnosed (19) with AL and had not undergone chemotherapy. Patients with other previous or concurrent malignant tumors or a history of known cardiomyopathy or congestive heart failure were excluded from this study. The AL group was subdivided according to the white blood cell (WBC) count into hyperleukocytic acute leukemia (HAL) and non-hyperleukocytic acute leukemia (NHAL). In this study, HAL was defined as a total WBC count > 100 × 10^9^/L (20). The CAD group included patients diagnosed (21) for the first time with no hematological diseases or other malignant tumors. The control group included healthy participants who had undergone a physical examination at the physical examination center, and none of them had heart-related diseases, leukemia, or other known cancers. Before starting the research, a written informed consent was signed by all study participants. All procedures followed the tenets of the Declaration of Helsinki and were approved by the Ethics in Research Committee of Wuhan University in Wuhan, China. This study was conducted and reported in accordance with the STROBE statement (22).

### Measurements and Data

Collected the data in the medical records of patients at the first visit: age (years), sex, cardiovascular risk factors (hypertension, diabetes, and smoking history), AL type, routine blood tests: white blood cells (WBCs, 10^9^/L), red blood cells (RBCs, 10^12^/L), hemoglobin (HGB, g/L), platelets (PLT, 10^9^/L), alanine aminotransferase (ALT, U/L), aspartate transaminase (AST, U/L), blood urea nitrogen (BUN, mmol/L), creatinine (Cr, μmol/L), D-dimer (DD, ng/ml), biochemical blood indicators: creatine kinase (CK, U/L), creatine kinase myocardial band (CK-MB, U/L), hydroxybutyrate dehydrogenase (HBDH, U/L), lactate dehydrogenase (LDH, U/L), highly sensitive troponin-I (hs-cTnI, pg/ml), B-type natriuretic peptide (BNP, pg/mL) and echocardiography parameters: left atrial internal diameter (LAID, mm), left ventricular internal diameter (LVID, mm), interventricular septal (IVS, mm), left ventricular posterior wall (LVPW, mm), end-diastolic volume (EDV, ml), end-systolic volume (ESV, ml), stroke volume (SV, ml) and ejection fraction (EF, %).

### Statistical Analysis

We performed linear regression analysis of biochemical blood indicators, myocardial enzymes, and echocardiographic indicators after logarithmic conversion. Normally distributed samples were compared using independent sample *t*-tests. The Mann-Whitney U rank-sum test was used for non-normally distributed samples and qualitative data were analyzed with the chi-square test. To reduce confounding in the AL group, such as anemia and cardiovascular risk factors, propensity score matching (PSM) analysis was conducted between the AL and control groups. The log-rank test was used to perform univariate analysis through Kaplan-Meier curves, and meaningful values were screened for multivariate Cox regression analysis. Odd ratios (ORs) and hazard ratios (HRs) were expressed with 95% confidence intervals (CIs). Statistical analysis was conducted using SPSS version 25.0 (IBM Corp., Armonk, NY, United States) and GraphPad Prism version 7.0 (GraphPad, San Diego, CA, United States). A value of *p* < 0.05 was considered statistically significant.

## Results

### Biochemical Blood Indicators and Echocardiography

**FIGURE 1:**
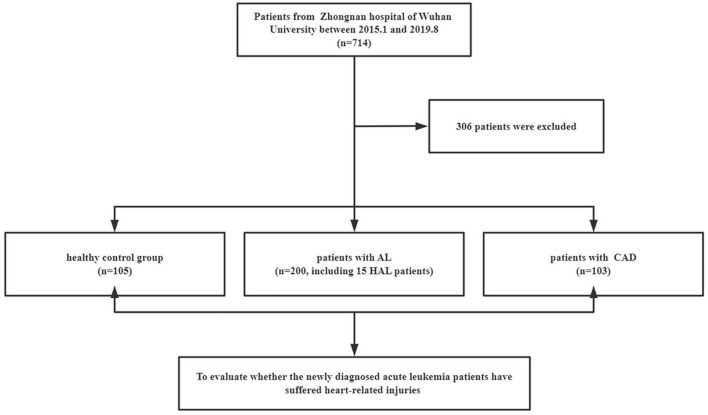
Selection of participants. AL, acute leukemia; CAD, coronary artery disease; HAL, hyperleukocytic acute leukemia.

A total of 408 individuals participated in this study, including 200 newly diagnosed AL patients (including 15 HAL patients), 103 patients with confirmed CAD, and 105 control patients. We eliminated patients who did not meet the study requirements. The screening flowchart is shown in Figure 1 and baseline patient characteristics are shown in Table 1. The mean age (range) of study participants in the control, AL, and CAD groups was 55 (44–63), 57 (41–66), and 62 (55–70) years, respectively. There were statistically significant differences in diabetes and routine blood indexes (WBC, RBC, HGB, PLT) between the AL and control groups. Meanwhile, there were significant differences in hypertension, diabetes, AST, BUN, and Cr between the CAD and control groups.

**TABLE 1:** Clinical characteristics of the study participants.

Characteristics	Control group	Patients with AL		Patients with CAD	
	*n* = 105	*n* = 200	*P*	*n* = 103	*P*
Age, year (range)	55 (44–63)	57 (41–66)	0.553	62 (55–70)	**<0.001**
**Sex (%)**					
*Male*	57 (54.3)	112 (56)	0.804	68 (66)	0.085
*Female*	48 (45.7)	88 (44)		35 (34)	
**Hypertension (%)**					
*Yes*	20 (19)	36 (18)	0.491	64 (62.1)	**<0.001**
*No*	73 (69.5)	164 (82)		39 (37.9)	
**Diabetes (%)**					
*Yes*	12 (11.4)	20 (10)	**0.002**	26 (25.2)	**<0.001**
*No*	93 (88.6)	180 (90)		77 (74.8)	
**Smoking History (%)**					
*Yes*	22 (21)	46 (23)	0.919	32 (31.1)	0.247
*No*	71 (67.6)	154 (77)		71 (68.9)	
Type (%)			–		–
*AML-M1*	–	10 (5)		–	
*AML-M2*	–	48 (24)		–	
*AML-M3*	–	20 (10)		–	
*AML-M4*	–	13 (6.5)		–	
*AML-M5*	–	28 (14)		–	
*ALL*	–	20 (10)		–	
*Uncategorized AL*	–	61 (30.5)		–	
WBC	0.834 ± 0.119	0.967 ± 0.716	**0.012**	0.803 ± 0.148	0.093
RBC	0.647 ± 0.058	0.366 ± 0.150	**<0.001**	0.626 ± 0.061	0.177
HGB	2.102 ± 0.047	1.871 ± 0.142	**<0.001**	2.010 ± 0.052	0.715
PLT	2.162 ± 0.085	1.519 ± 0.456	**<0.001**	2.268 ± 0.119	0.740
ALT	1.279 ± 0.229	1.278 ± 0.326	0.992	1.327 ± 0.281	0.176
AST	1.309 ± 0.132	1.345 ± 0.308	0.157	1.421 ± 0.303	**0.001**
BUN	0.723 ± 0.131	0.710 ± 0.196	0.478	0.777 ± 0.156	**0.008**
Cr	1.821 (1.749–1.872)	1.786 (1.708–1.880)	0.077	1.821 (1.742–1.895)	**<0.001**

*Data are expressed as mean ± SD, median (interquartile range), or number (percentage). Bold values indicate statistical significance. AL, acute leukemia; AML, acute myeloid leukemia; ALL, acute lymphoblastic leukemia; ALT, alanine aminotransferase; AST, aspartate transaminase; BUN, blood urea nitrogen; CAD, coronary artery disease; Cr, creatinine; HGB, hemoglobin; PLT, platelet; RBC, red blood cell; WBC, white blood cell.*

**FIGURE 2:**
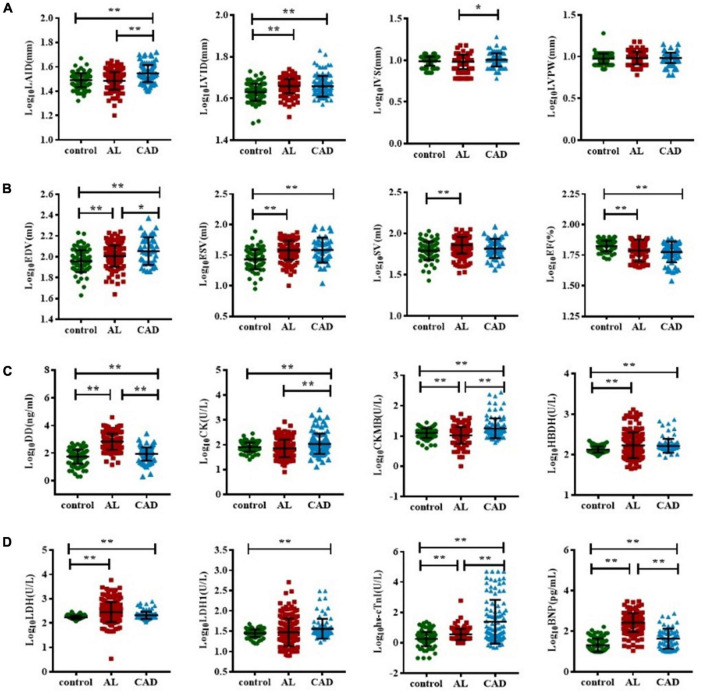
Comparison of cardiac function impairment indicators in acute leukemia, coronary artery disease, and control group patients. The *T* test was used to compare the myocardial enzyme and echocardiography indexes of the three group **(A–D)**. (*represents *p* < 0.05, ** represents *p* < 0.01). BNP, B-type natriuretic peptide; CK, creatine kinase; CK-MB, creatine kinase myocardial band; DD, D-dimer; EDV, end-diastolic volume; ESV, end-systolic volume; EF, ejection fraction; hs-cTnI, highly sensitive troponin-I; HBDH, hydroxybutyrate dehydrogenase; IVS, interventricular septal; LVPW, left ventricular posterior wall; LDH, lactate dehydrogenase; LAID, left atrial internal diameter; LVID, left ventricular internal diameter; SV, stroke volume.

A comparison of cardiac damage index results among the three groups is shown in Figure 2. The mean values of biochemical blood indicators, such as DD, CK-MB, HBDH, LDH, hs-cTnI, and BNP and echocardiography indexes such as LVID, EDV, ESV, and SV were significantly higher, while EF was significantly lower, in the AL group than in the control group (*p* < 0.05). In the comparison between the control and CAD groups, it could be found that the CAD group had more severe myocardial damage. The average values of echocardiography parameters LAID, LVID, EDV, and ESV were significantly higher, and EF value was significantly lower, in the CAD group compared with the control group; the average values of cardiac function and myocardial enzyme indexes increased significantly. Comparing the AL and the CAD groups, the average values of LAID, IVS, EDV, CK, CK-MB, and hs-cTnI in the CAD group were significantly higher, while only the values of DD and BNP were higher in the AL group.

The indicators with significant differences between the AL and control group were included in the multivariate linear regression analysis, and the multiple linear regression equation was fitted. The model had 60.3% explanatory power (*R*^2^ = 0.603) for whether the AL group had damage from heart disease. Using multiple linear stepwise regression analysis, the final factors entering the equation were CK-MB, LDH, BNP, DD, and LVID (*p* < 0.05; Table 2).

**TABLE 2:** Multivariate association of clinically relevant indicators between AL and control groups (related variables).

Risk factors	Beta	*t*	*P*	OR (95% CI)
CK-MB	−0.102	−2.763	**0.006**	0.903 (0.708–0.944)
HBDH	0.005	0.108	0.914	NA^a^
LDH	0.131	3.448	**0.001**	1.140 (1.083–1.339)
BNP	0.535	12.502	**<0.001**	1.707 (1.423–1.624)
hs-cTnI	0.003	0.084	0.933	NA^a^
DD	0.238	5.942	**<0.001**	1.269 (1.090–1.186)
LVID	0.149	3.806	**<0.001**	1.161 (2.430–16.281)
EDV	−0.042	−0.903	0.367	NA^a^
ESV	0.067	1.412	0.159	NA^a^
SV	−0.013	−0.279	0.780	NA^a^
EF	−0.041	0.108	0.914	NA^a^

*Multivariable association R = 0.603 for entire model. Bold values indicate statistical significance. 95% CI, OR and P values based on a multiple linear stepwise regression model; Reported P values are adjusted to control false discovery rate. BNP, B-type natriuretic peptide; CK-MB, creatine kinase myocardial band; CI, confidence interval; DD, D-dimer; EDV, end-diastolic volume; ESV, end-systolic volume; EF, ejection fraction; hs-cTnI, highly sensitive troponin-I; HBDH, hydroxybutyrate dehydrogenase; LDH, lactate dehydrogenase; LVID, left ventricular internal diameter; NA, not available; OR, odds ratio; SV, stroke volume. ^a^OR and P values not reported due to limited observations.*

### Propensity Score Matching Analysis

A total of 44 patients with AL and 44 control patients (1:1) were matched for age, sex, cardiovascular risk factors (hypertension, diabetes, and smoking history), and hemoglobin concentration. The indexes with significant differences between the matched AL and control group were included in the multiple linear regression analysis. Using multiple linear stepwise regression analysis, the final factors entering the equation were BNP, HBDH, and LVID (*p* < 0.05; Tables 3, 4).

**TABLE 3:** Clinical and echocardiographic characteristics of matched AL and control group patients.

Variable	Matched control group	Matched patients with AL	*P*
	*n* = 44	*n* = 44	
Age, year (range)	55 (40–63.8)	51 (34.8–64.5)	0.544
**Sex (%)**			
*Male*	23 (52.3)	28 (63.6)	0.286
*Female*	19 (43.2)	16 (36.4)	
**Hypertension (%)**			
*Yes*	8 (18.2)	6 (13.6)	0.565
*No*	36 (81.8)	38 (86.4)	
**Diabetes (%)**			
*Yes*	0 (0)	0 (0)	–
*No*	0 (0)	0 (0)	
**Smoking history (%)**			
*Yes*	6 (13.6)	8 (18.2)	0.565
*No*	38 (86.4)	36 (81.8)	
WBC	0.826 ± 0.128	1.057 ± 0.745	0.049
RBC	0.651 ± 0.060	0.546 ± 0.073	**<0.001**
HGB	2.045 ± 0.035	2.040 ± 0.056	0.587
PLT	2.177 ± 0.085	1.684 ± 0.471	**0.001**
ALT	1.303 ± 0.209	1.428 ± 0.352	0.503
AST	1.311 ± 0.126	1.345 ± 0.318	0.026
BUN	0.740 ± 0.155	0.720 ± 0.215	0.620
Cr	1.811 (1.731–1.904)	1.807 (1.669–1.869)	0.060
DD	1.789 ± 0.544	2.486 ± 1.221	**<0.001**
CK	1.907 ± 0.206	1.845 ± 0.293	0.256
CK-MB	1.133 ± 0.147	1.102 ± 0.270	0.512
HBDH	2.133 ± 0.079	2.244 ± 0.355	**0.045**
LDH	2.234 ± 0.084	2.582 ± 0.487	**<0.001**
LDH1	1.452 ± 0.088	1.549 ± 0.380	0.104
hs-cTnI	0.302 ± 0.522	0.452 ± 0.180	0.075
BNP	1.398 ± 0.408	2.281 ± 0.452	**<0.001**
LAID	1.491 ± 0.055	1.480 ± 0.066	0.423
LVID	1.628 ± 0.033	1.654 ± 0.039	**0.001**
IVS	1.000 (0.952–1.000)	1.000 (0.954–1.041)	0.676
LVPW	1.000 (0.954–1.000)	0.982 (0.954–1.041)	0.138
EDV	1.974 ± 0.102	1.989 ± 0.111	0.522
ESV	1.455 ± 0.151	1.550 ± 0.173	**0.007**
SV	1.819 ± 0.094	1.841 ± 0.108	0.313
EF	1.834 ± 0.042	1.804 ± 0.051	**0.004**

*Data are expressed as mean ± SD, median (interquartile range), or number (percentage). Bold values indicate statistical significance. Bold values indicate statistical significance. The two groups were matched 1:1 with age, sex, cardiovascular risk factors and hemoglobin number, and the caliper value was set to 0.2. ALT, alanine aminotransferase; AST, aspartate transaminase; BNP, B-type natriuretic peptide; BUN, blood urea nitrogen; CK, creatine kinase; CK-MB, creatine kinase myocardial band; Cr, creatinine; DD, D-dimer; EDV, end-diastolic volume; EF, ejection fraction; ESV, end-systolic volume; HBDH, hydroxybutyrate dehydrogenase; HGB, hemoglobin; hs-cTnI, highly sensitive troponin-I; IVS, interventricular septal; LAID, left atrial internal diameter; LDH, lactate dehydrogenase; LVID, left ventricular internal diameter; LVPW, left ventricular posterior wall; NA, not available; OR, odds ratio; PLT, platelet; RBC, red blood cell; SV, stroke volume; WBC, white blood cell.*

**TABLE 4:** Multivariate correlation of clinically relevant indicators between matched AL and control group patients (related variables).

Risk factors	Beta	*t*	*P*	OR (95% CI)
BNP	0.648	8.434	**<0.001**	1.912 (1.496–1.919)
LDH	0.113	1.079	0.284	NA^a^
HBDH	0.200	2.689	**0.009**	1.221 (1.105–1.952)
LVID	0.174	2.224	**0.029**	1.190 (1.275–76.861)
ESV	−0.175	−0.800	0.075	NA^a^
EF	0.066	0.787	0.434	NA^a^

*Multivariable association R = 0.553 for entire model. Bold values indicate statistical significance. 95% CI, OR and P values based on multiple linear stepwise regression model; Reported P values are adjusted to control false discovery rate. BNP, B-type natriuretic peptide; CI, confidence interval; EF, ejection fraction; ESV, end-systolic volume; HBDH, hydroxybutyrate dehydrogenase; HGB, hemoglobin; LDH, lactate dehydrogenase; LVID, left ventricular internal diameter; OR, odds ratio. ^a^OR and P values not reported due to limited observations.*

### Subgroup Analysis

**FIGURE 3:**
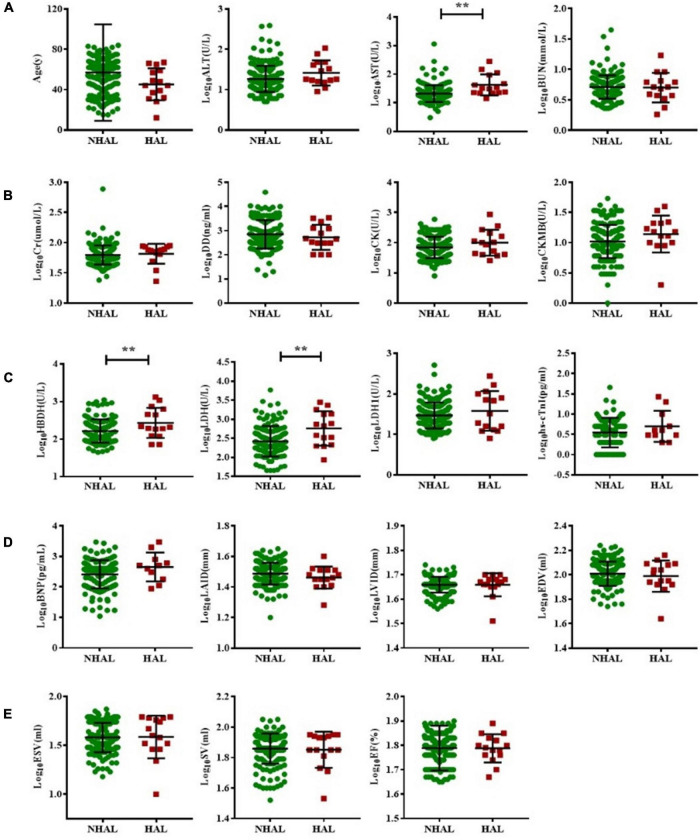
Subgroup analysis of non-hyperleukocytic and hyperleukocytic acute leukemia groups. The *T* test was used to compare BNP and myocardial enzyme and echocardiography indexes between the NHAL HAL groups **(A–E)**. (** represents *p* < 0.01). AST, aspartate transaminase; ALT, alanine aminotransferase; BNP, B-type natriuretic peptide; BUN, blood urea nitrogen; Cr, creatinine; CK, creatine kinase; CK-MB, creatine kinase myocardial band; DD, D-dimer; EDV, end-diastolic volume; ESV, end-systolic volume; EF, ejection fraction; hs-cTnI, highly sensitive troponin-I; HBDH, hydroxybutyrate dehydrogenase; HAL, hyperleukocytic acute leukemia; IVS, interventricular septal; LVPW, left ventricular posterior wall; LDH, lactate dehydrogenase; LAID, left atrial internal diameter; LVID, left ventricular internal diameter; NHAL, non-hyperleukocytic acute leukemia; SV, stroke volume.

Acute leukemia patients were subdivided into HAL or NHAL groups according to the WBC count. We found that AST, HBDH, and LDH were significantly higher in the HAL group than in the NHAL group (*p* < 0.05). There were no significant differences between the two groups in terms of other serological indicators or echocardiogram-related indicators (Figure 3).

### Prognostic Analysis

**FIGURE 4:**
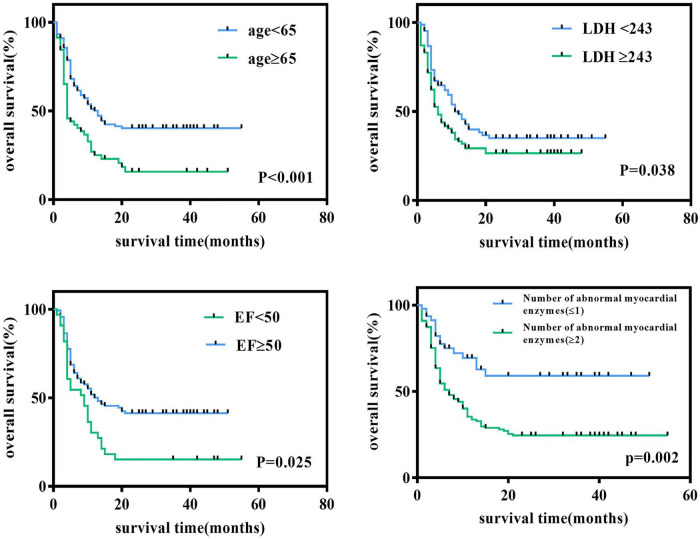
Survival curve of newly diagnosed acute leukemia patients. Log-rank test for univariate analysis of heart-related indicators in newly diagnosed acute leukemia patients through Kaplan-Meier curves. LDH, lactate dehydrogenase; EF, ejection fraction.

**FIGURE 5:**
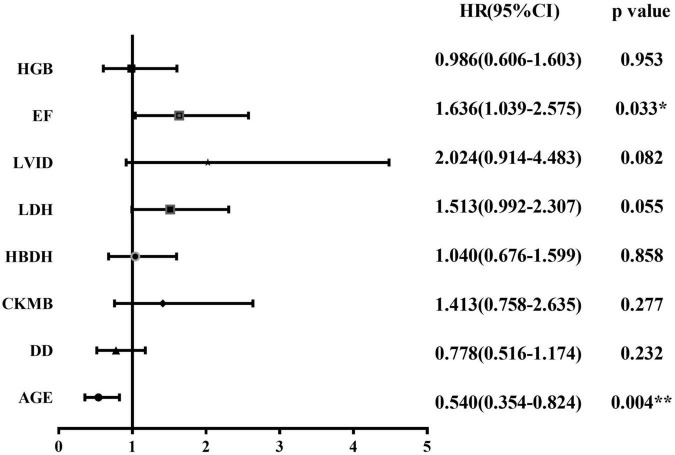
Forest plot for Cox regression analysis. Cox regression model for multivariate analysis. *p < 0.05, **p < 0.01. HRs and 95% CIs are estimated in Cox proportional hazards regression. CI, confidence interval; CK-MB, creatine kinase myocardial band; DD, D-dimer; EF, ejection fraction; HBDH, hydroxybutyrate dehydrogenase; HGB, hemoglobin; HRs, hazard ratios; LDH, lactate dehydrogenase; LVID, left ventricular internal diameter.

We followed all newly diagnosed AL patients for prognostic analysis. The median follow-up time was 35 months (range 1–55). We analyzed the relationship among age, sex, cardiovascular risk factors (hypertension, diabetes, and smoking history), biochemical blood indicators, myocardial enzyme indexes, echocardiography indexes, and the number of abnormal myocardial enzyme indexes and survival, to screen for risk factors related to the prognosis of the disease. Variables with statistically significant effect measurement values included age (*p* < 0.001), LDH (*p* = 0.038), EF (*p* = 0.025), and the number of myocardial enzyme abnormalities (*p* = 0.002). The meaningful survival curve is shown in Figure 4. In addition, the meaningful variables or close to meaningful values (*p* < 0.1) in the previous single-factor analysis were subjected to multi-factor Cox regression analysis. The results showed that age (HR = 0.540, 95%CI: 0.354–0.824, *p* = 0.004) and EF (HR = 1.636, 95% CI: 1.039–2.575, *p* = 0.033) were independent factors related to the prognosis of AL patients (Figure 5).

### ROC Curve

**FIGURE 6:**
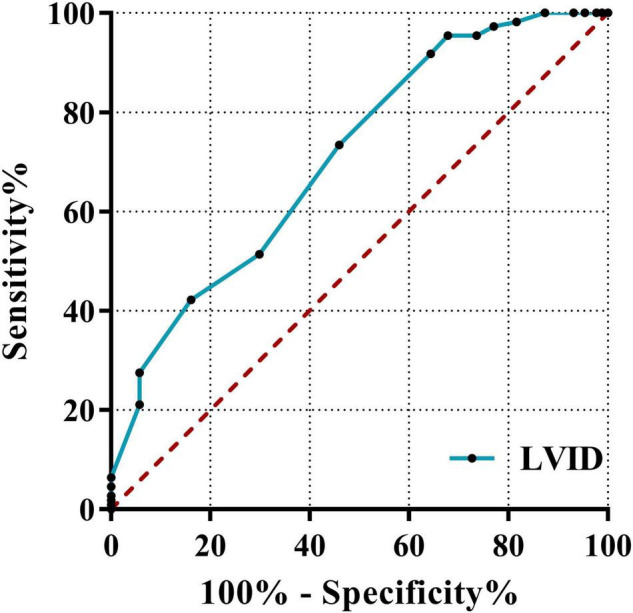
Receiver operating characteristic curves of LVID. The ROC curve using the clinical diagnosis of cardiac function as the dependent variable and myocardial enzymes, BNP, and echocardiography indicators as independent variables. LVID, left ventricular internal diameter; ROC, receiver operating characteristic; AUC, area under the curve.

Classification based on the echocardiography clinical diagnosis in newly diagnosed AL patients include significant differences in myocardial enzymes and related echocardiographic indicators; the three indicators with the highest results for the ROC area under the curve (AUC) were LVID (0.709), EDV (0.675), and HBDH (0.591). This result revealed that the best predictor of leukemic myocardial damage was LVID. It showed the predictive ability of LVID (≥43.5 cm) (AUC = 0.709, 95%CI: 0.637–0.781, *p* < 0.001; sensitivity = 95.4%; specificity = 32.2%). The ROC curve is shown in Figure 6.

### Pearson Correlation Analysis

Pearson correlation analysis was performed for multiple variables related to the all-AL cases. The result of this analysis revealed that the percentage of bone marrow blasts was positively correlated with DD (*r* = 0.440), CK (*r* = 0.361), hs-cTnI (*r* = 0.433), and BNP (*r* = 0.379), while negatively correlated with EF (*r* = −0.257). Furthermore, WBC count was positively correlated with HBDH, LDH, and LDH1. LVID was positively correlated with age, CK, LDH, LDH1, EDV, ESV, and SV and negatively correlated with HGB and EF (Supplementary Table 1).

## Discussion

This study examined the correlation between clinical characteristics, cardiac biomarkers, and cardiac imaging parameters in newly diagnosed AL patients. The results showed that compared with the control group, AL patients had already experienced some heart-related injuries, accompanied by a significant increase in CK-MB, LDH, hs-cTnI, BNP, and LVID, and a decrease in EF. However, the degree of cardiac damage was less than that of the CAD group. PSM analysis showed that cardiac damage in AL patients was significantly manifested in BNP, HBDH, and LVID. Subgroup analysis showed that cardiac damage was more severe in the HAL than NHAL subgroup. Furthermore, the best predictor of cardiac damage in AL patients was LVID, while age and EF were independent prognostic risk factors. The ratio of bone marrow blasts was positively correlated with the index of cardiac damage.

In recent years, cardiac tumors have been recognized as an important aspect of cardio-oncology practice where diagnosis and management are critical (23, 24). However, most scholars only focus on heart damage caused by anti-tumor therapy (25, 26). Cardiotoxicity during the treatment of AL patients has been extensively reported (27), including radiotherapy (28, 29), conventional chemotherapies (e.g., anthracyclines, antimetabolites, and cyclophosphamide) (30–32), immune checkpoint inhibitors (programmed cell death 1, programmed death ligand 1, or CTL-associated protein 4) (33, 34), and many targeted therapies, particularly monoclonal antibodies and tyrosine kinase inhibitors (35, 36), CART therapy (37–39), natural killer cell immunotherapy (40), and hematopoietic stem cell transplantation (41–43). Possible mechanisms are mediated by reactive oxygen species generated in iron-dependent chemical reactions (44), mitochondrial dysfunction (45), increased calcium flux in cardiomyocytes (46), and disorders of DNA topoisomerase 2-beta metabolism (47). However, only a small amount of research has been done on cardiac lesions directly attributed to leukemia in AL patients.

Patients with leukemia can have secondary cardiac tumors. It has been reported that cardiac tumors metastasize most frequently from the respiratory system, followed by the hematopoietic system (leukemia/multiple myeloma), and most commonly involve the pericardium, followed by myocardium, epicardium, and endocardium (14, 47, 48). Cardiac infiltration is frequently observed in leukemia patients at autopsy, with an incidence of 11–44% (48–52). However, most patients with cardiac infiltrates are clinically asymptomatic, and only a small number of cases presented pericardial effusion, hypertrophic cardiomyopathy, or AMI (15, 49, 53–59) as the first sign. There appears to be no significant difference in these microanatomical or clinical features between AML and ALL patients. Therefore, we have reason to speculate that AL itself will cause the heart lesions, and anti-cancer treatment may be an aggravating factor for cardiotoxicity. However, due to the concealment of clinical symptoms in patients with newly diagnosed AL, the incidence of cardiac dysfunction reported in the current study may only be the tip of the iceberg. Furthermore, as these cases occur more frequently in older patients with cardiac risk factors, the role of AL itself on heart disease may be overlooked.

Our analysis of cardiac-related parameters in newly diagnosed AL patients showed that compared with controls, AL patients did experience some cardiac-related lesions, both in myocardial enzymes and echocardiographic parameters. These clinical manifestations are similar to existing reports and recommendations for cardiac management monitoring in cancer patients (60–62). However, at the time of diagnosis, AL patients often present with anemia and increased WBC count that can cause a certain degree of myocardial damage, which introduces confounding factors to our research results. However, it should be noted that the WBCs of AL patients contain many leukemia cells, which differs from the WBCs of other diseases. Therefore, we matched the AL with the control group and tried to adjust for possible confounding factors, including age, sex, cardiovascular risk factors (hypertension, smoking), and hemoglobin, and found that cardiac injury in the AL group remained. In a subgroup comparison of HAL and NHAL, we found that HAL caused more severe myocardial enzymatic changes. This may be related to the stagnation and accumulation of leukemia cells (58, 63, 64). In addition, correlation analysis showed that the proportion of blast cells was positively correlated with heart damage. Although the correlation coefficient was not particularly high, it still suggested that leukemia itself may cause direct damage to the heart.

Acute leukemia disease itself may be associated with cardiac damage because it shares systemic pathogenic pathways and mechanisms with CVDs: inflammation, metabolic alterations, clonal hematopoiesis, angiogenesis alterations, and extracellular matrix and stroboscopic cells (13, 65). Cancer metabolic byproducts can also impair cardiac function. An increased amount of d-2-hydroxyglutaric acid produced by IDH2-mutant leukemia cells directly contributes to cardiac insufficiency by inhibiting α-KGDH (66). Furthermore, cancer cells can directly secrete factors that induce cardiomyocyte atrophy and metabolic changes, but the exact signaling pathways in cardiomyocytes remain poorly understood (67).

For the reasons described above, clinicians should comprehensively evaluate patients’ cardiac function before chemotherapy. Echocardiography can be used to observe changes in LVEF (68). Conditioned individuals can examine peak systolic global longitudinal strain by speckle-tracking echocardiography (69), which can detect cardiotoxicity at an early stage. In addition, cardiac biomarkers (26) such as troponin, high-sensitivity troponin, BNP, cardiac enzymes, and other potential biomarkers such as those for endothelial dysfunction, myocardial ischemia, oxidative stress and inflammation should be evaluated for a comprehensive assessment to achieve early real-time identification, assessment, and monitoring of cardiotoxicity, and timely targeted treatment. In addition, some studies (26, 70) suggest that early application of cardioprotective therapy to cancer patients may be considered, but its role has not been proven or recommended.

There were several limitations in this study. First, in order to eliminate confounding factors, we used PSM analysis, but the sample size after PSM analysis was small, which may cause some data bias and be different from the real-world situation. Second, we focused on the systolic function of the ventricle, and did not collect related indicators of ventricular diastolic function, thus possibly missing some correlations. Finally, this was a single-center observational study and the sample size was small. Larger sample size validation and prospective studies are needed to confirm the degree of cardiac damage in patients with leukemia. Nonetheless, our study provides important information about the relationship between cardiac damage and leukemia.

In summary, we identified significant differences in cardiac function between patients with primary AL and control patients, and these differences could have an impact on patient prognosis. Our findings provide new insights into the complex etiology and molecular events leading to cardiac injury in patients with AL, and how they can alert clinicians to the presence of cardiac injury in AL patients. In addition, more research is still needed to confirm and explore the specific mechanism between leukemia and cardiac lesions.

## Data Availability Statement

The raw data supporting the conclusions of this article will be made available by the authors, without undue reservation.

## Ethics Statement

The studies involving human participants were reviewed and approved by the Ethics Research Committee of Wuhan University in Wuhan, China. The patients/participants provided their written informed consent to participate in this study.

## Author Contributions

WX, LM, YT, and FZ conceived and designed this research. LM, FY, GC, and JW were responsible for collecting the clinical outcome data of the patients included in the study. YL and TR re-checked the data. WX, YS, QW, and NZ performed the statistical analysis. WX, LM, and FZ wrote the manuscripts and created figures and tables. All authors strictly reviewed the manuscript and approved the final submitted manuscript.

## Conflict of Interest

The authors declare that the research was conducted in the absence of any commercial or financial relationships that could be construed as a potential conflict of interest.

## Publisher’s Note

All claims expressed in this article are solely those of the authors and do not necessarily represent those of their affiliated organizations, or those of the publisher, the editors and the reviewers. Any product that may be evaluated in this article, or claim that may be made by its manufacturer, is not guaranteed or endorsed by the publisher.
